# *BvCPD* promotes parenchyma cell and vascular bundle development in sugar beet (*Beta vulgaris* L.) taproot

**DOI:** 10.3389/fpls.2023.1271329

**Published:** 2023-09-12

**Authors:** Xiaotong Guo, Yue Li, Ningning Li, Guolong Li, Yaqing Sun, Shaoying Zhang

**Affiliations:** Sugar Beet Physiological Research Institute, Inner Mongolia Agricultural University, Hohhot, China

**Keywords:** brassinosteroid, BvCPD gene, sugar beet, transgenic plant, parenchyma cell, vascular bundle development

## Abstract

Constitutive photomorpogenic dwarf (*CPD*) is a pivotal enzyme gene for brassinolide (BR) synthesis and plays an important role in plant growth, including increasing plant biomass and plant height, elongating cells, and promoting xylem differentiation. However, little is known about the function of the *CPD* gene in sugar beet. In the current study, we isolated *CPD* from *Beta vulgaris* L. (*BvCPD*), which encodes protein localized in the nucleus, cell membrane, and cell wall. *BvCPD* was strongly expressed in parenchyma cells and vascular bundles. The transgenic sugar beet overexpressing *BvCPD* exhibited larger diameter than that of the wild type (WT), which mainly owing to the increased number and size of parenchyma cells, the enlarged lumen and area of vessel in the xylem. Additionally, overexpression of *BvCPD* increased the synthesis of endogenous BR, causing changes in the content of endogenous auxin (IAA) and gibberellin (GA) and accumulation of cellulose and lignin in cambium 1–4 rings of the taproot. These results suggest that *BvCPD* can promote the biosynthesis of endogenous BR, improve cell wall components, promote the development of parenchyma cells and vascular bundle, thereby playing an important role in promoting the growth and development of sugar beet taproot.

## 1 Introduction

Sugar beet (*Beta vulgaris* L.), a biennial plant in the Chenopodiaceae family, is the most important raw material in the production of sucrose (Ramesh et al., 2017). The fleshy taproot is the main harvest portion of the beet and is rich in sucrose. Sugar production occurs via the leaves through photosynthesis during the first year of growth. The sugar is continuously transferred from leaves to the taproots and stored in concentric rings of vascular tissue originating from the secondary cortex of early root development as well as in parenchyma cells that increase in number and become larger during growth (Rae et al., 2005). Therefore, understanding the regulatory mechanisms underlying taproot growth will enable the development new sugar beet cultivars with high yield and quality.

Brassinolide (BR) is an important plant hormone and plays an critical role in regulating many biological processes, including root development, cell division and elongation, vascular bundle differentiation, pollen fertility, photomorphology, and stress resistance processes (Wei and Li, 2016; Nolan et al., 2020; Chen et al., 2022). Specifically, the mechanism by which BR regulates root growth and lateral development has been extensively studied in a variety of plants, including *Arabidopsis thaliana* (Vukašinović et al., 2021), *Oryza sativa* L (Huang et al., 2022), *Triticum aestivum* L (Avalbaev et al., 2021; Xu et al., 2022), *Solanum lycopersicum* (Bai et al., 2021), *Phyllostachys pubescens* (Wang et al., 2019; Guo et al., 2020), *Populus* L (Tian et al., 2018; Feng et al., 2021), *Eriobotrya japonica* (Su et al., 2021), and *Beta vulgaris* (Zhang et al., 2017; Wang et al., 2021). For example, mutants associated with BR biosynthesis or signaling genes, such as *Arabidopsis dwf7-1*, *bri1*, and *bin2*, cause abnormal xylem differentiation ratios and defects in vascular bundle development compared with the wild type (WT), suggesting that BRs can regulate vascular structure and xylem formation. (Choe et al., 1999; Caño-Delgado et al., 2004). Exogenous spraying of BR promoted the development of parenchyma cell and secondary xylem in sugar beet taproot and regulated the expression of cell wall biosynthesis-related genes, *BvXTH33, BvSHV3, BvCESA6, BvPARVUS*, and *BvCEL1*, which affected the diameter of taproot (Wang et al., 2021). In *Liriodendron*, BR treatment significantly increased the length of fibers and vascular elements, and induced the biosynthesis of cellulose and hemicellulose (Hyunjung et al., 2014). This indicates that BR has a regulatory role in the biosynthesis and modification of secondary cell wall components and cell wall assembly during secondary xylem development in woody plants. Furthermore, BR is most abundant in actively growing tissues. Unlike other plant hormones, BR is not transported over long distances and acts only near the site of synthesis (Symons et al., 2008). Therefore, research on BR biosynthesis and its role in modulating root growth and development can provide a reference for regulating sugar beet taproot development using molecular biology or chemical control.

The biosynthesis of BR is controlled by multiple genes, most of which encode cytochrome P450 (*CYP90* or *CYP85*), such as *CPD*, *DWF4*, *DET2*, and *BR6OX2*. The *CPD* gene encodes a C-3 oxidase and plays a vital role in multiple steps of BR synthesis. The CPD protein sequence of guayule rubber particles has all the functional domains of microsomal cytochrome P450 monooxygenase, including an N-terminal cell membrane anchoring sequence, a proline-rich region, and a region for oxygen and hemoglobin binding. Furthermore, the *CPD* gene has a specific sterol hydroxylase domain (Pan et al., 1995). *AtCPD* can catalyze early BR intermediates i.e., (22S)-22-hydroxy campesterol, (22R,23R)-22,23-dihydroxycampesterol, 6-deoxocathasterone, and 6-deoxoteasterone; however, it is most potent in the catalysis of (22S)-22-hydroxy campesterol (Ohnishi et al., 2012). *CPD* has the function and necessity for plant growth and is widely expressed in root, stalk, leaf, and inflorescence (Mathur et al., 1998; Wang et al., 2015; Vukašinović et al., 2021). The function of this gene has been extensively studied in a variety of plants. *Arabidopsis cpd* mutants exhibit a yellowing phenotype under dark conditions, with hypocotyls becoming shorter, no apical curved grooves, cotyledons opening, leaf primordia appearing, and photoinduced genes no longer being inhibited. Under light conditions, it exhibits a dwarf and male sterile phenotype (Miklós et al., 1996). *OsCPD* regulates the biosynthesis of BR in *Oryza sativa* L, resulting in enlarged leaf angles and increased grain size; furthermore, it also plays a significant and redundant role in maintaining plant structure (Zhan et al., 2022). Overexpression of the potato *CPD* gene strengthens the ability of seedlings to remove reactive oxygen species and reduce oxidative damage by increasing BR content, enhancing antioxidant enzyme activity and improving plant resistance to PEG-induced osmotic stress, thereby affecting plant growth. (Zhou et al., 2016). Overexpression of *PeCPD* enhances BR signaling, increase plant height and biomass, promote xylem differentiation, and reduced pith size, thereby improving wood quality (Feng et al., 2021). Moreover, *PeCPD* regulates BL synthesis, allowing BL to interact with growth hormones and partially replace the action of cytokinin to stimulate healing tissue formation (Tian et al., 2018). When *CPD* is knocked down, cell growth in the meristem and elongation regions of the roots is inhibited, leading to root shortening (Vukašinović et al., 2021). However, there is little research on the function of the *CPD* gene in the radial development of plant roots, which needs to be further explored.

Our previously published transcriptome sequencing results showed that the *Bv_qyup* gene in the *CPD* family is closely related to sugar beet taproot enlargement (Zhang et al., 2017). A total of 76 *BvCPD* family members were detected in a sugar beet database, clustered into 10 subgroups and distributed on 9 chromosomes. The *Bv_qyup* gene is located on chromosome 2 and encodes the CPD protein. Overexpression of this gene in *Arabidopsis thaliana* showed phenotypes of root elongation, increased root volume, enlarged leaves, and increased length of fruit pods, which may be important in improving plant development (Zhu et al., 2020). However, the sugar beet root belongs to a typical tritrophic structure, and it has not been reported how this gene plays a role in regulating taproot growth and development. Herein, we investigated in depth the tissue expression pattern of *BvCPD* (*Bv_qyup*) in sugar beet, constructed overexpression and RNAi lines using agrobacterium-mediated genetic transformation, and elucidated the mechanism of *BvCPD* in regulating the growth and development of sugar beet taproots through morphological, anatomical, and molecular biological studies. The research confirms for the first time that the function of *CPD* in sugar beets, providing candidate genes for genetic improvement of high yield and quality in sugar beet. Simultaneously, we created high yield planting resources for sugar beet and provided a scientific basis for chemical regulation of yield traits in sugar beets.

## 2 Materials and methods

### 2.1 Plant material

We used high-sugar homozygous sugar beet variety BS02, cultivated by the Institute of Sugar Beet Physiology, Inner Mongolia Agricultural University. The growth environment of the histoculture and artificial climate chamber was 22°C, 60% humidity, and 16/8 h light/dark cycles.

### 2.2 Subcellular localization of BvCPD

Coding sequences (CDS) of *BvCPD* were obtained by PCR amplification using gene-specific primers BvCPD-G. PCR products (1,468 bp) were digested with Kpn I and Sal I, then cloned into the pCAMBIA1300-35S-EGFP vector. The plasmids of the fusion vector and empty vector were extracted and transferred into agrobacterium tumefaciens GV3101 receptor cells to infect the onion epidermis.

Onion phosphorus stems were sterilized and washed in sterile water on an ultra-clean bench, cut lengthways, and the thicker inner layer of the middle scale-leaf was taken. Using a scalpel, we gently cut several 1 cm^2^ squares, tore off epidermal cells, and placed them in MS medium and incubated them at 25°C for 24 h. Then, we took 3.3 mg of gold powder and placed it in a centrifuge tube containing 55 µL of 40% PEG. We took 20 µl and add 5 μg of 35S-BvCPD-EGFP or 35S-EGFP plasmid DNA, 50 µL of 2.5 M CaCl_2_, 8 µL of 0.1 M Spermidine. This was fully mixed and centrifuged for 30 s, and the supernatant was discarded. The remained was rinsed once with 70% ethanol, and we then add 15 µL of absolute ethanol before vortex and mixing. Then, 15 μL of the mixture was placed on the carrier membrane to dry. The cultured onion epidermal cells were placed in a vacuum chamber for 30 h to rupture the membrane. The onion was incubated at 25°C in the dark for 48 h. In addition, onion epidermal cells were treated with 0.3 g/mL sucrose solution for plasmolysis and then imaged. Each onion epidermal cell was removed and placed on a slide for observation and image acquisition using a fluorescent microscope (OLYMPUS, U-RFL-T, Japan).

### 2.3 Fluorescence *in situ* hybridization in paraffin sections of sugar beet

Beet roots were removed, washed, cut crosswise, and immediately placed in fixative for 24 h. A vacuum pump was used to evacuate air; thereafter, dehydration by gradient alcohol was followed by wax immersion and embedding. The paraffin was sliced by a slicer, retrieved with slice spreaders, and baked at 62°C for 2 h. Sections were then placed in dewaxing clear solution I and II for 15 min each, and anhydrous ethanol I and II for 5 min each. The samples were dried spontaneously and then immersed in DEPC water. Proteinase K (20ug/ml; Servicebio; Article number: G1234) was added dropwise and digested at 37°C for 25 min. We then added pre-hybridization solution (Servicebio; Article number: G3016-3) and incubated at 37°C for 1 h before discarding the liquid. We added the probe CPD-ORF hybridization solution (Synthesized by Servicebio, China) to a concentration of 500 nM and hybridized overnight at 42°C. The probe information is presented in **Supplementary Table S1**. Next, the sections were washed with eluent and incubated with DAPI (4’,6-diamidino-2-phenylindole) for 8 min in the dark before mounting. Finally, the sections were placed under a fluorescent microscope (OLYMPUS, U-RFL-T, Japan) for observation and image acquisition.

### 2.4 Vector construction and genetic transformation of sugar beet

Construction of *BvCPD* overexpression vector: The CDSs of *BvCPD* were acquired by PCR amplification using gene specific primers BvCPD-OE. PCR products (1,523 bp) were digested with Bam H I and Pst I, then cloned into the ToPopBoLn-TA vector. We selected the positive colonies with correct sequencing to extract the recombinant plasmid, cut BvCPD with Bam H I and Pst I double enzymes, recovered the digested product by agarose gel electrophoresis, and inserted the T4 ligase into the plant expression vector pCAMBIA1300-35S-X cut by the corresponding restriction enzyme. In this way, we successfully constructed a plant overexpression vector containing the complete CDS region of the *BvCPD* gene for genetic transformation into sugar beet.

Construction of *BvCPD* silencing vector: CDSs of *BvCPD* were acquired by PCR amplification using gene specific primers BvCPD-RP and BvCPD-RA. PCR products (334 bp) were digested with Bam H I and Spe I, Kpnl, and Sac I, then cloned into the pEASYR-Blunt Zero Cloning Vector. We selected the positive colonies with correct sequencing and extracted the recombinant plasmid using BamH I/Spe I double enzyme digestion for *BvCPD* forward fragments, and KpnI/SacI double enzyme digestion for *BvCPD* reverse fragments. After recovering the enzymatic cleavage products, T4 ligase was inserted into the plant expression vector pCAMBIA1300-35S-X after corresponding restriction endonuclease cleavage. In this way, we constructed a plant silencing expression vector containing both positive and negative fragments of the *BvCPD* gene.

Planting sterile seedlings: Seeds were washed for 30 min, treated with 0.1% HgCl_2_ solution for 10 min, flushed 3–4 times with sterile water, dried on filter paper for 5–10 min, and planted into a germination medium. Agrobacterium-mediated genetic transformation of sugar beet: We cut the sprouted beet cotyledonary node to form a wound, pre-cultured for 24 h, overnight cultured the agrobacterium containing the target gene to an OD value of 0.6–0.8, centrifuged and resuspend to 0.5 with suspension, and dipped the beet cotyledon node for 10 min. Excess agrobacterium was blotted dry on filter paper and incubated on a co-culture medium for 2–3 days in the dark. Inhibiting agrobacterium growth: After 2–3 days, we washed the cotyledonary nodes 3–4 times and transferred them to a suppressive medium for 5–7 days. Screening for positive plants: We transferred the sterile beet cotyledons after inhibition to a screening medium containing Temetin, and after 15–20 days transferred the screened beet resistant shoots to a differentiation medium for differentiation and expansion. Promoting root growth: When sugar beet seedlings reached 3–5 cm, they were transferred to rooting medium to induce root formation. Transplanting: After 1 month, the beet seedlings were differentiated based on thick roots and leaves topping out to the cap; we opened the cap and refined these seedlings for 3–5 days, transplanted them into seedling trays (nutrient soil: vermiculite = 3:1), covered the box, and cultivate in the dark for 2–3 days (**Supplementary Figure S1**). The composition of all the media used is presented in **Supplementary Table S2**.

### 2.5 Phenotypic determination of sugar beet

#### 2.5.1 Phenotype

Morphological indicators of sugar beet were measured when transplanted transgenic sugar beet seedlings were incubated in an artificial climate chamber for 80 days. Three plants were taken from each line. Fresh weight per plant, leaf plexus weight, fresh leaf weight, petiole weight, root weight, taproot weight, and fibrous root weight were weighed on an electronic balance; plant height, leaf length, leaf width, root length, and taproot length were measured with a tape measure; the diameter of the taproot was measured with a vernier caliper.

#### 2.5.2 Anatomic structure

Beet roots were cut to a thickness of 5 mm along the first leaf scar after 80 days of transplant culture and placed in FAA fixative for 24 h and dehydrated. Next, 12-μm thick transverse sections were obtained using a rotary slicer, stained with astra blue and saffron, and then fixed on paraffin (Maia et al., 2018). The samples were observed under a microscope and micrographs were taken. Images were analyzed using the CaseViewer software. We assessed the following histological parameters: ring spacing, area of parenchyma cells, number of parenchyma cell layers, area of xylem, and area of vessel lumen. The distance between the outermost xylem of two vascular forming layers was defined as the spacing between the rings of forming layers; the area of 10 adjacent parenchyma cells was selected to measure the area; the number of parenchyma cell layers between the rings of two forming layers was counted; the xylem area per unit area was counted using CaseViewer magnified to 47x field of view; and the area of the ductal pore lumen on the xylem of each forming layer was counted. All lines had three biological replicates.

### 2.6 Hormone determination

We sampled sugar beet roots by inserting a punch diagonally from the outside to the inside. Three plants from each line were taken. Plant tissue was ground under liquid nitrogen freezing conditions, and 0.1 g of fresh sample was mixed with 900 μL of 0.1% PBS buffer. After sufficient shaking, the tissue was centrifuged at 12,000 rpm for 30 min in a cryogenic freezing centrifuge. The supernatant was taken for hormone determination. BR, IAA, and GA contents were determined by enzyme-linked immunoassay (ELISA) according to the manufacturer’s instructions (Jiangsu Su Enzyme Science and Technology Co., Ltd.).

### 2.7 Gene expression identification by qRT–PCR

Total RNA was extracted according to the operating instructions of the TransZol Up Plus RNA Kit (ER501). RNA was reverse transcribed to complementary DNA (cDNA) using TransScript One-Step gDNA Removal and cDNA Synthesis Super Mix (AT311), and diluted 5-fold for use. Experiments were performed using the CFX96 Real-Time Quantitative PCR System (Bio-Rad, Hercules, CA, USA). The reaction system included 10 μL of PerfectStartTM Green qPCR SuperMix (AQ601), upstream and downstream primers (10 μM) of 0.4 μL each, 7.2 μL of nuclease-free water, and 2 μL of cDNA template. The PCR amplification procedure was 30 s at 94°C, followed by 5 s at 94°C, 15 s at 55°C, and 30 s at 72°C for a total of 45 cycles, with only one product amplified and detectable after each reaction for melt curve analysis. The 2^-ΔΔCT^ method was used to calculate gene relative expression (Livak and Schmittgen, 2001). Three biological replicates and three technical replicates were taken for the experimental samples. Primer sequences are shown in **Supplementary Table S3**.

### 2.8 Cellulose and lignin quantification

After 120 days of transplantation of sugar beet, samples were taken from the vascular rings and inter-rings of the 1–4 layers of the taproot, cut into fine shreds to dry and then ground into powder. Determination of cellulose and lignin content was performed according to the manufacturer’s instructions (Jiangsu Addison Biotechnology Co., China).

### 2.9 Data analysis

Differences between means were assessed using SPSS (VER) at the 5% and 1% probability levels. Statistical plots of the data were produced using GraphPad Prism.

## 3 Results

### 3.1 BvCPD is localized in the nucleus, cell membrane, and cell wall

**Figure 1:**
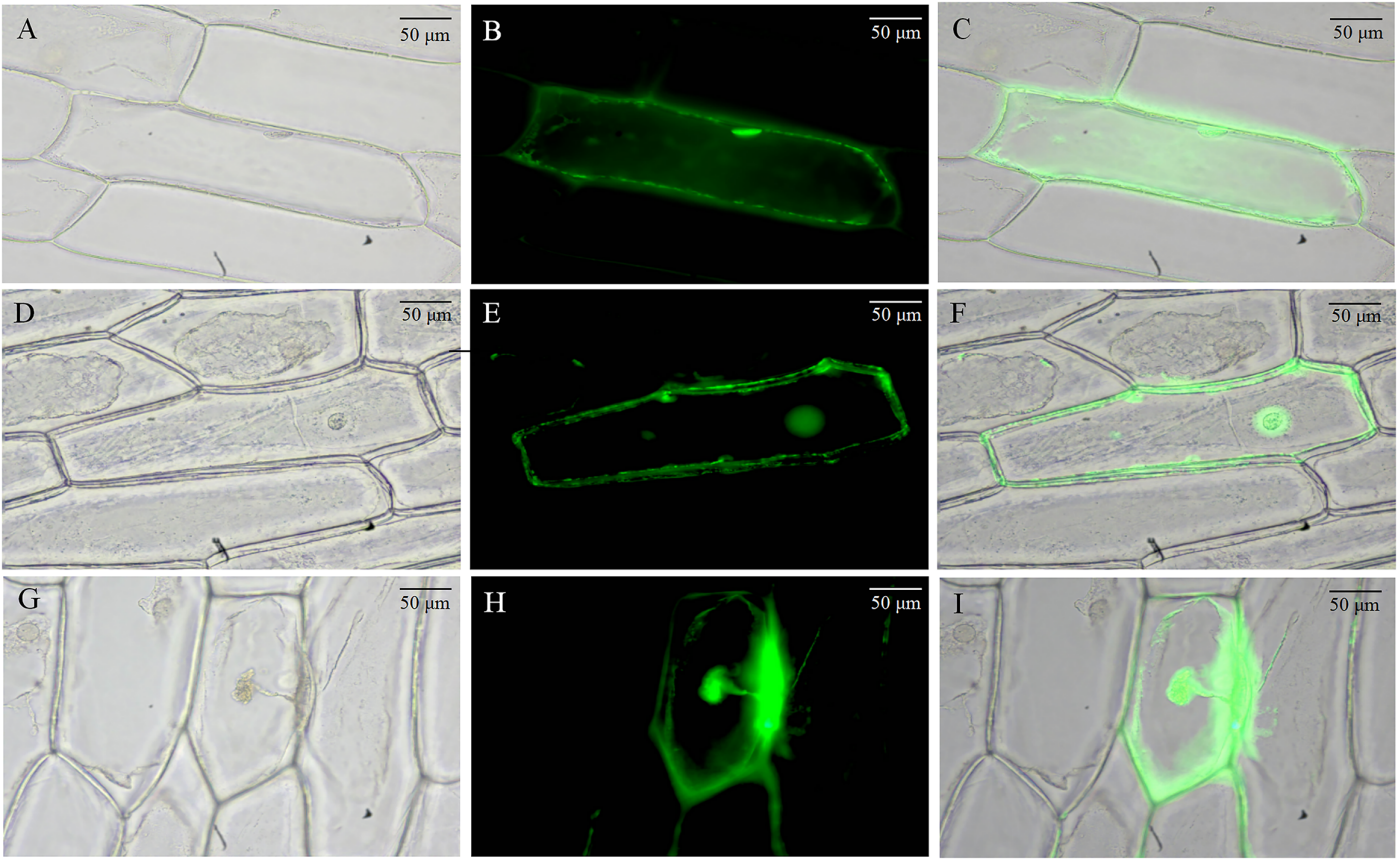
Subcellular localization of BvCPD in onion epidermis. **(A–C)** Subcellular localization of 35S-EGFP. **(D–F)** Subcellular localization of 35S-BvCPD-EGFP before plasmolysis. **(G–I)** Subcellular localization of 35S-BvCPD-EGFP after plasmolysis. Scale bars = 50 μm.

We used the onion epidermis as a model for subcellular localization of BvCPD proteins. The 35S-BvCPD-EGFP fusion expression vector and 35S-EGFP empty vector were genetically transformed into onion epidermal cells for transient expression by particle bombardment. Fluorescence microscopy observations showed that the control 35S-initiated EGFP protein signal was distributed throughout the onion epidermal cells (**Figures 1A–C**). The BvCPD protein was expressed in the nucleus, cell membrane, and cell wall (**Figures 1D–I**).

### 3.2 *BvCPD* genes are strongly expressed in sugar beet parenchyma cells and vascular bundles

**Figure 2:**
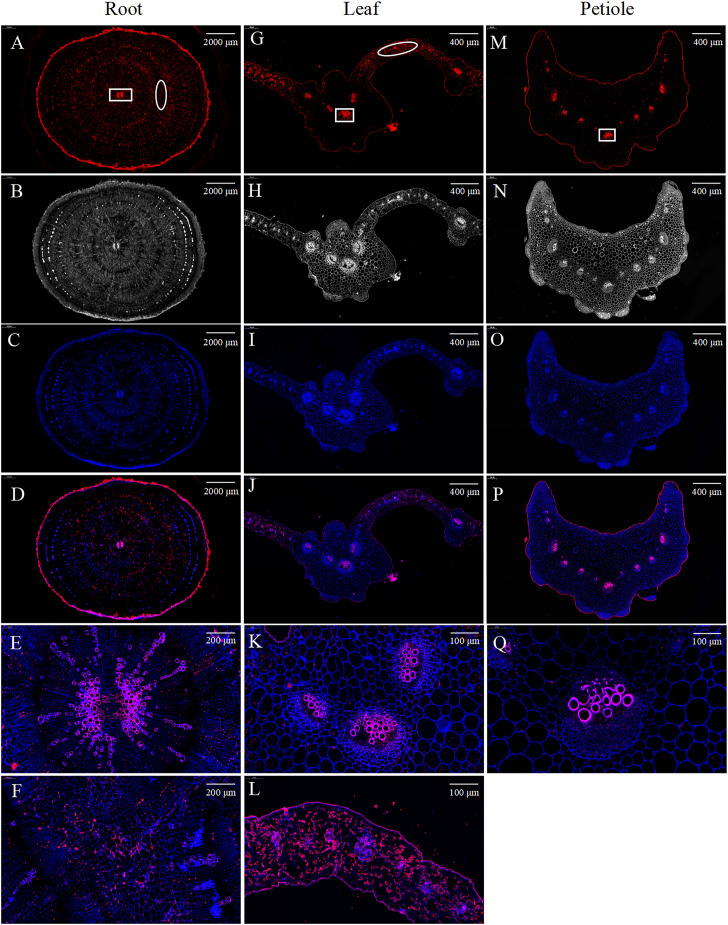
Fluorescence *in situ* hybridization of *BvCPD* in sugar beet. **(A–D)** Root. Scale bar = 2,000 μm **(E)** Enlarged area of root, denoted by the square in **(A)**, representing the vascular bundle. Scale bar = 200 μm. **(F)** Enlarged area of root, denoted by the oval **(A)**, representing parenchyma cells. Scale bar = 200 μm. **(G–J)** Leaf. Scale bar = 400 μm **(K)** enlarged area of leaf, denoted by the square in **(G)**. Scale bar = 100 μm. **(L)** Enlarged area of leaf, denoted by the oval in **(G)**. Scale bar = 100 μm. **(M–P)** Petiole. Scale bar = 400 μm. **(Q)** Enlarged area of petiole, denoted by the square in **(M)**. Scale bar = 100 μm. **(A, G, M)** Red images showing *BvCPD* expression regions. **(B, H, N)** Blue images showing cell wall autofluorescence. **(C, I, O)** Bright field images. **(D, J, P)** Composite images.

mRNA fluorescence *in situ* hybridization results showed that *BvCPD* was mainly expressed in cell walls of inter-ring parenchymal cells and in the xylem of vascular bundles in root cross sections (**Figures 2A–F**). In leaf cross sections, *BvCPD* was mainly expressed in vascular bundles and mesophyll cells, with strong expression in the xylem of vascular bundles (**Figures 2G–L**). In petiole cross sections, *BvCPD* was expressed in the vascular bundle, with high expression in the xylem and low expression in the phloem (**Figures 2M–Q**). These results suggest that the *BvCPD* gene may play an essential role in the development of parenchymal cells and vascular bundles in sugar beet.

### 3.3 Acquisition of *BvCPD* transgenic sugar beet plants

**Figure 3:**
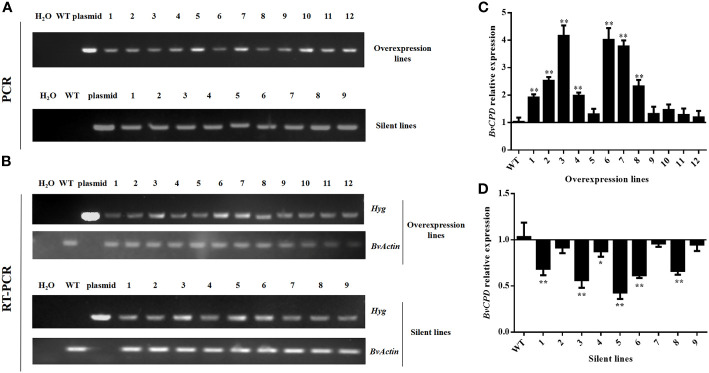
Identification of transgenic lines. **(A)** Electrophoresis plots of PCR products of the *Hyg* gene of the overexpressing and silent lines. **(B)** Electrophoresis plot of the RT–PCR products of the *Hyg* gene of the overexpressing and silent lines. **(C)** Relative expression of *BvCPD* in the overexpression lines. **(D)** Relative expression of *BvCPD* in silent lines. _*_Student’s-t test, P < 0.05; _**_ Student’s-t test, P < 0.01.

The optimized *Agrobacterium tumefaciens* genetic transformation system was used to impregnate cotyledon nodes of sugar beet. After screening for hygromycin (6 mg/L) resistance, 21 lines with hygromycin resistance were obtained, including 12 overexpression-resistant lines and 9 silenced-resistant lines. Polymerase chain reaction (PCR) with primer HYG-2 was performed using DNA from resistant plants as the template, and the expected target gene products (**Figure 3A**) were obtained in all the cases, confirming the insertion of exogenous genes in the genomes of these plants. The lines were subjected to mass expansion, rooting, and transplanting. Reverse transcription–polymerase chain reaction (RT–PCR) results showed that transcripts of the screening gene hygromycin (*Hyg*) were detected in all the lines of the transgenic sugar beet, confirming that the target gene was already stably expressed (**Figure 3B**). Quantitative real–time polymerase chain reaction (qRT–PCR) technology was used to detect the expression of *BvCPD* in transgenic lines. The results showed that compared with WT, the expression level of *BvCPD* was increased in overexpressed lines, but decreased in silent lines (**Figures 3C, D**). Due to the different insertion sites of target gene, the expression levels varied among different lines. The three lines with the highest differences in expression levels as compared to WT were selected from overexpressed and silent lines, and named OE1, OE2, OE3 and R1, R2, and R3, respectively, for subsequent research.

### 3.4 *BvCPD* can promote the growth of sugar beet

**Figure 4:**
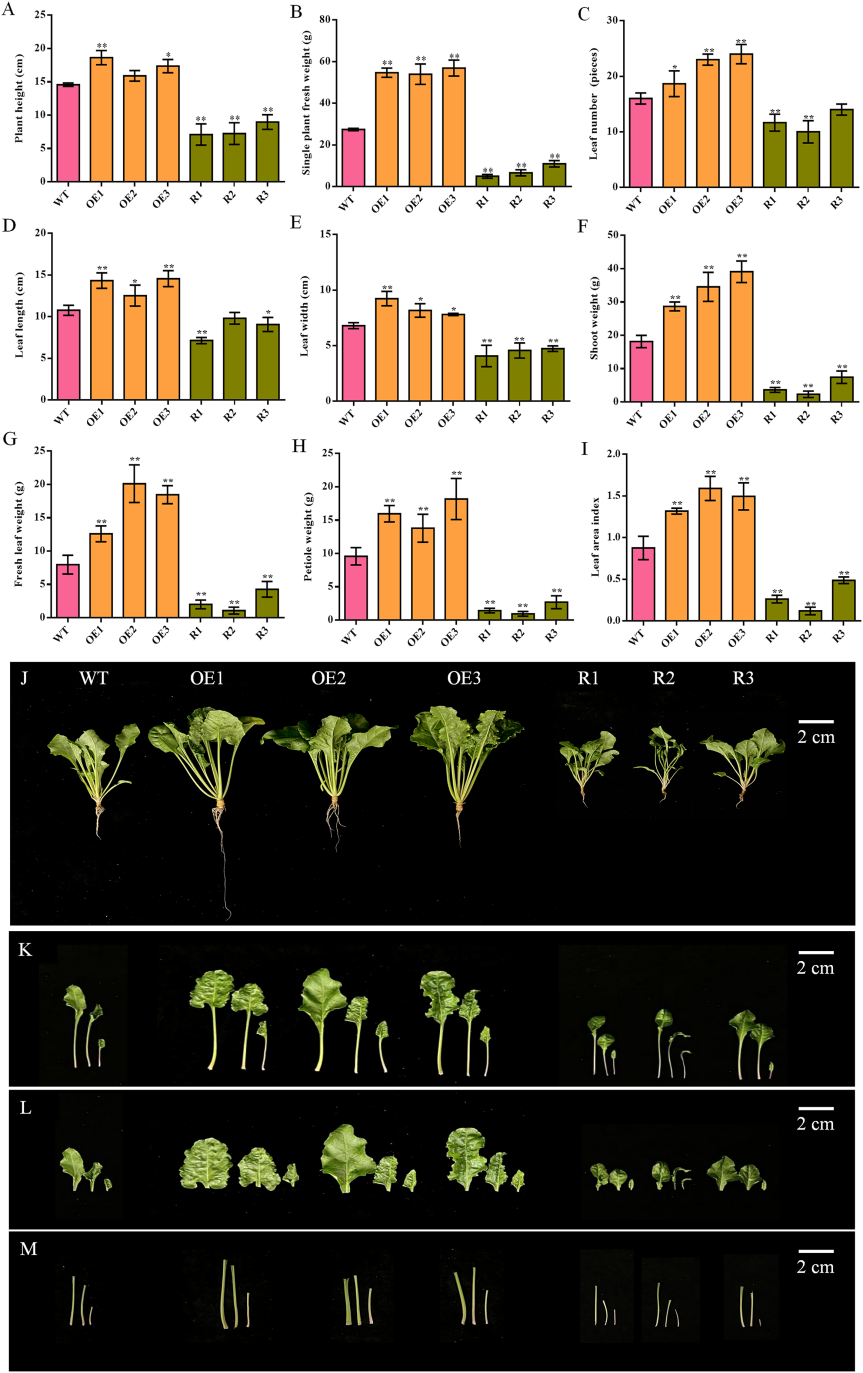
Phenotype determination of the aerial part between transgenic sugar beet and WT. **(A)** Plant height. **(B)** Single plant fresh weight. **(C)** Leaf number. **(D)** Leaf length. **(E)** Leaf width. **(F)** Shoot weight. **(G)** Fresh leaf weight. **(H)** Petiole weight. **(I)** Leaf area index. **(J)** Phenotype of each transgenic line and WT 1 month after transplanting. **(K)** Phenotype of the aerial part. **(L)** Phenotype of leaf. **(M)** Phenotype of petiole. _*_Student’s t-test, P < 0.05; _**_Student’s-t test, P < 0.01. Scale bars = 2 cm.

The shoot morphologies of the different transgenic lines and the wild type varied significantly. The morphological indicators of each leaf bush of the transgenic plants overexpressing *BvCPD* were significantly greater than those of WT, while those of silent lines were significantly smaller (**Figures 4A–I**). Compared with WT, the increase in leaf fresh weight of the overexpressed lines was greater than that of other indicators, increasing by 57.92%–152.24% (**Figure 4G**); leaf length and leaf width increased by 16.41%–35.29% and 14.71%–35.78%, respectively (**Figures 4D, E**). Petiole was significantly thickened (**Figure 4M**). Silent lines had a slender petiole (**Figure 4M**) and the largest reduction in petiole weight (decrease of 71.89%–90.08%; **Figure 4H**). Leaf length and leaf width decreased by 8.98%–33.75% and 30.39%–40.20%, respectively. In summary, the overexpression of *BvCPD* facilitates more vigorous growth of the shoots (**Figures 4J–M**).

**Figure 5:**
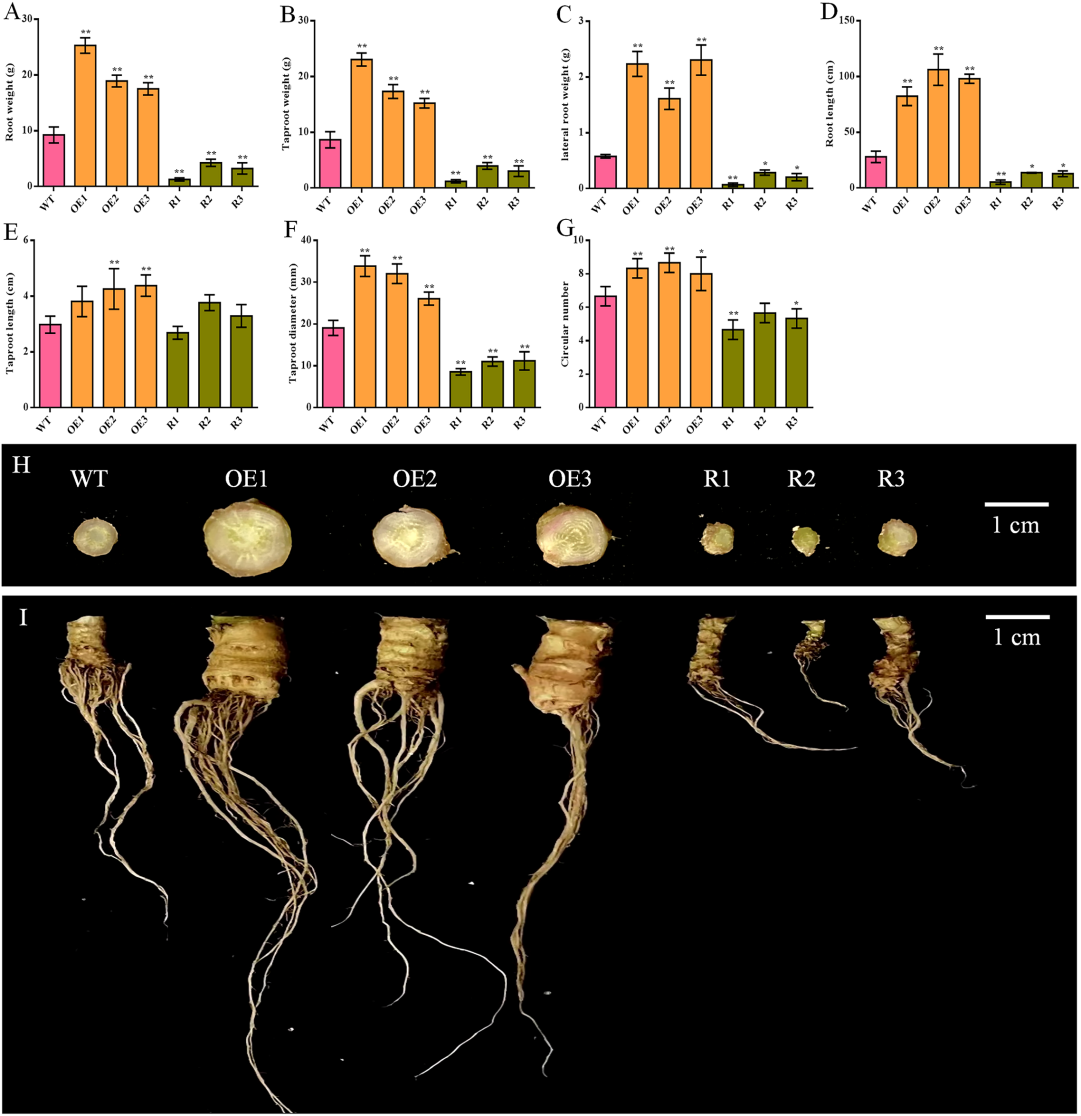
Phenotype determination of the underground parts of transgenic sugar beet and WT. **(A)** Root weight. **(B)** Taproot weight. **(C)** Fibrous root weight. **(D)** Root length. **(E)** Taproot length. **(F)** Taproot diameter. **(G)** Circular number. **(H)** Taproot cross-section of each transgenic line and WT. **(I)** Phenotype of the roots of transgenic lines and WT 1 month after transplanting. _*_ Student’s-t test, P < 0.05; _**_ Student’s-t test, P < 0.01. Scale bars = 1 cm.

Comparing the root of sugar beet from various lines, all indicators were significantly increased in *BvCPD* overexpression lines and significantly decreased in silent lines, except for taproot length, for which there was no significant difference in most lines (**Figures 5A–G**). The taproot weight and lateral root weight of the overexpression lines were the most significantly different, increasing by 75.70%–166.01% and 178.16%–297.70%, respectively as compared to WT; silent lines decreased by 54.17%–85.89% and 50.57%–87.93%, respectively (**Figures 5B, C**). In addition, taproot diameters of overexpressed and silenced lines increased and decreased by 36.80%–77.58% and 41.27%–55.08%, respectively (**Figures 5F, I**). Circular numbers increased and decreased by 1–2 compared with WT (**Figure 5G**). In summary, overexpression of *BvCPD* promotes taproot development (**Figures 5H, I**) by increasing the circular number of the taproot formation layer and the taproot diameter.

### 3.5 *BvCPD* expression is highest in the roots of transgenic sugar beet

**Figure 6:**
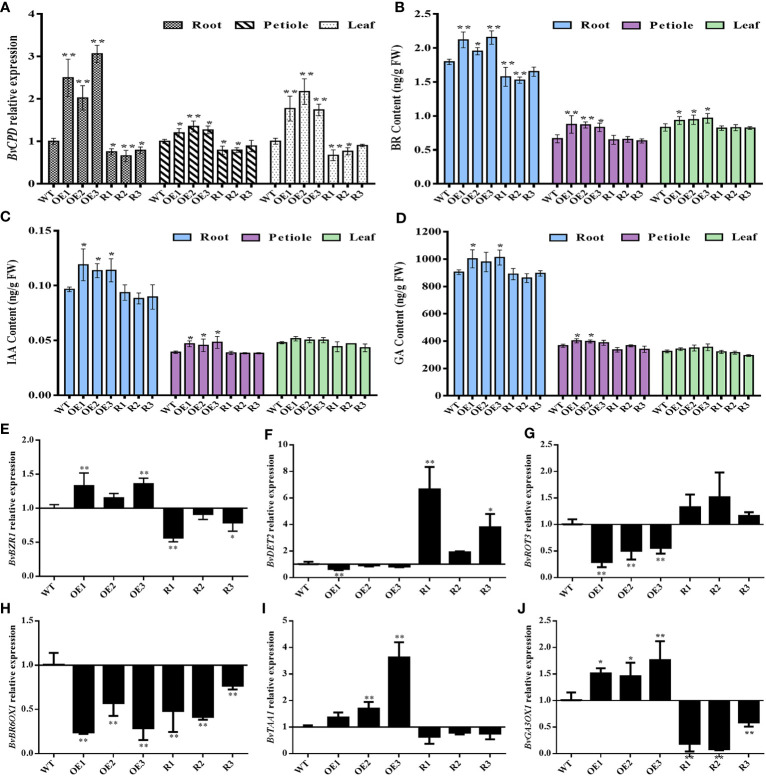
Determination of gene expression levels and hormone content in different transgenic lines and WT. **(A)** Relative expression of *BvCPD* in the root, petiole, and leaf of transgenic type and WT sugar beet. **(B–D)** BR, IAA, and GA content in root, petiole, and leaf. **(E–J)** Relative expression levels of *BvBZR1*, *BvDET2*, *BvROT3*, *BvBR6OX1*, *BvTAA1*, and *BvGA3OX1* in the root. _*_ Student’s-t test, P < 0.05; _**_ Student’s-t test, P < 0.01.

qRT–PCR was used to detect the relative expression of *BvCPD* in the roots, petiole, and leaf of transgenic beets to clarify expression changes in various organs. The expressions of *BvCPD* in the root, petiole, and leaf of each overexpressed and silenced line were significantly higher and lower than those of WT, respectively. *BvCPD* expression in the roots of overexpressed lines was greater than those in the leaf and petiole (**Figure 6A**). Among the three overexpression lines, OE3 had the highest expression level in the root. R2 was the silent line with the lowest expression level of *BvCPD* in the root.

### 3.6 *BvCPD* increases BR content and sugar beet root growth & development by cooperating with IAA and GA

To verify whether *BvCPD* plays a role in sugar beet BR synthesis, transgenic sugar beet BR content was measured. The BR contents in the root, petiole, and leaf of each overexpression line were significantly higher than those of WT, while for silent lines they were significantly lower, especially in the root (**Figure 6B**). Hormones IAA and GA, which are related to BR, interact to promote plant growth and development (Li et al., 2021). Therefore, endogenous IAA and GA in various organs were also analyzed. The variation trend of IAA and GA content in transgenic lines was consistent with BR, but the difference in BR content was more significant compared with WT (**Figures 6C, D**). This indicates that *BvCPD* can increase the BR content. Endogenous BR may play a leading role in the simultaneous mobilization of IAA and GA, which synergistically controls the growth and development of sugar beet, especially the root.

To analyze expression changes of genes related to BR, IAA, and GA synthesis and regulation in sugar beet, real-time fluorescence quantitative analysis was used to determine the brassinazole-resistant (*BvBZR*), de-etiolated2 (*BvDET2*), rotundifolia3 (*BvROT3*), BR-6-oxidase (*BvBR6OX1*), L-Tryptophan aminotransferase1 (*BvTAA1*), and gibberellin 3-oxidase1 (*BvGA3OX1*) genes. The expression of the BR regulatory gene *BvBZR1* was significantly upregulated in *BvCPD* overexpression lines, while the expression of BR synthesis genes *BvDET2* and *BvROT3* was downregulated. In silent lines, the expression of *BvBZR1* was downregulated, while *BvDET2* and *BvROT3* were upregulated. In contrast, the expression level of *BvBR6OX1* was downregulated in both overexpressed and silenced lines. *BvTAA1* and *BvGA3OX1*, as IAA and GA related synthetase genes, were upregulated in overexpressed lines, and downregulated in each silent line (**Figures 6E–J**). The results indicate that *BvCPD* is involved in the regulation of BR, IAA, and GA biosynthesis.

### 3.7 *BvCPD* promotes parenchyma cell enlargement, xylem development, and ductal pore lumen area

**Figure 7:**
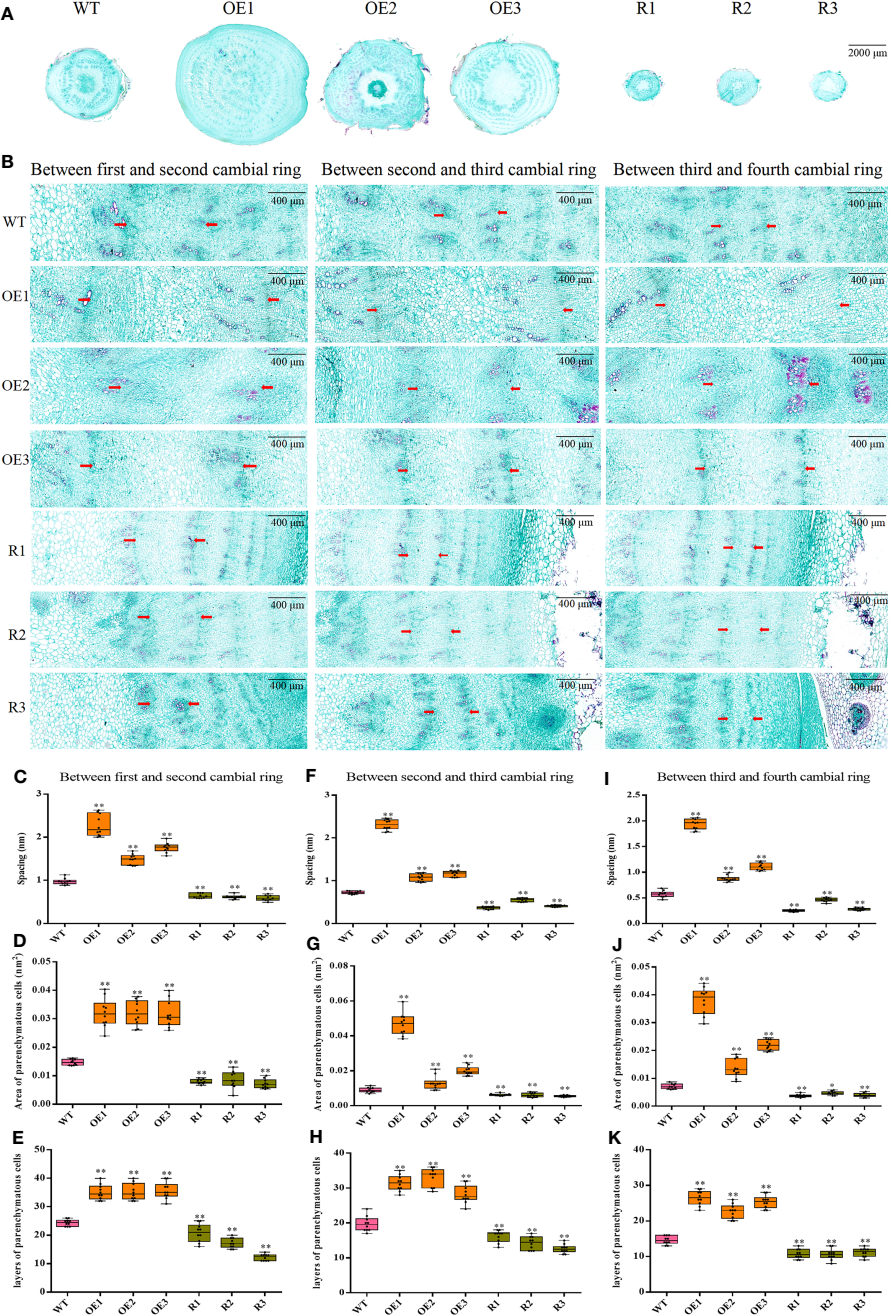
Determination of ring spacing, parenchyma cell size, and cell layer number in the first four rings. **(A)** Cross-section of wild type and transgenic sugar beet taproot. Scale bar = 2,000 μm. **(B)** First four ring-forming layers of different lines. Scale bar = 400 μm. **(C–E)** Spacing, area of parenchyma cells, and layers of parenchyma cells between the first and second cambial rings. **(F–H)** Spacing, area of parenchyma cells, and layers of parenchyma cells between the second and third cambial rings. **(I–K)** Spacing, area of parenchyma cells, and layers of parenchyma cells between the third and fourth cambial rings. _*_ Student’s-t test, P < 0.05; _**_ Student’s-t test, P < 0.01.

To assess how taproot structure affects taproot diameter, we examined 1–4 rings of each transgenic line taproot and WT (**Figures 7A, B**). Compared with WT, the ring spacing of the 1–2 formation layer of taproot was significantly increased in *BvCPD* overexpression lines. Ring spacing of OE1 is greatest in overexpression lines (2,275.39 μm). In contrast, there was an extremely significant reduction in ring spacing for silent lines, with the ring spacing of R3 being the smallest (587.84 μm; **Figure 7C**). Parenchymal cells between the 1–2 cambial rings of *BvCPD* overexpressed beet taproot were significantly larger than that of WT. The parenchymal cell area of OE2 was the largest in the overexpression lines (32,097.844 μm^2^). Parenchymal cells of RNAi silencing lines were significantly smaller than that of WT. RNAi silenced line R1 had the smallest parenchymal cell area (7,209.375 μm^2^; **Figure 7D**). Moreover, the amount of cell layers in all overexpressed lines increased significantly. The cell layer with the highest number was OE3, reaching 36 layers, which is 12 layers more than WT. The silence lines were significantly reduced, and the number of layers in R3 was half that of WT (**Figure 7E**). Similar results were found for the 2–3 and 3–4 cambium rings. In summary, *BvCPD* increases the size of parenchyma cells and number of layers in the first and second formation layer rings, thereby increasing the diameter of beet taproot (**Figures 7F–K**).

**Figure 8:**
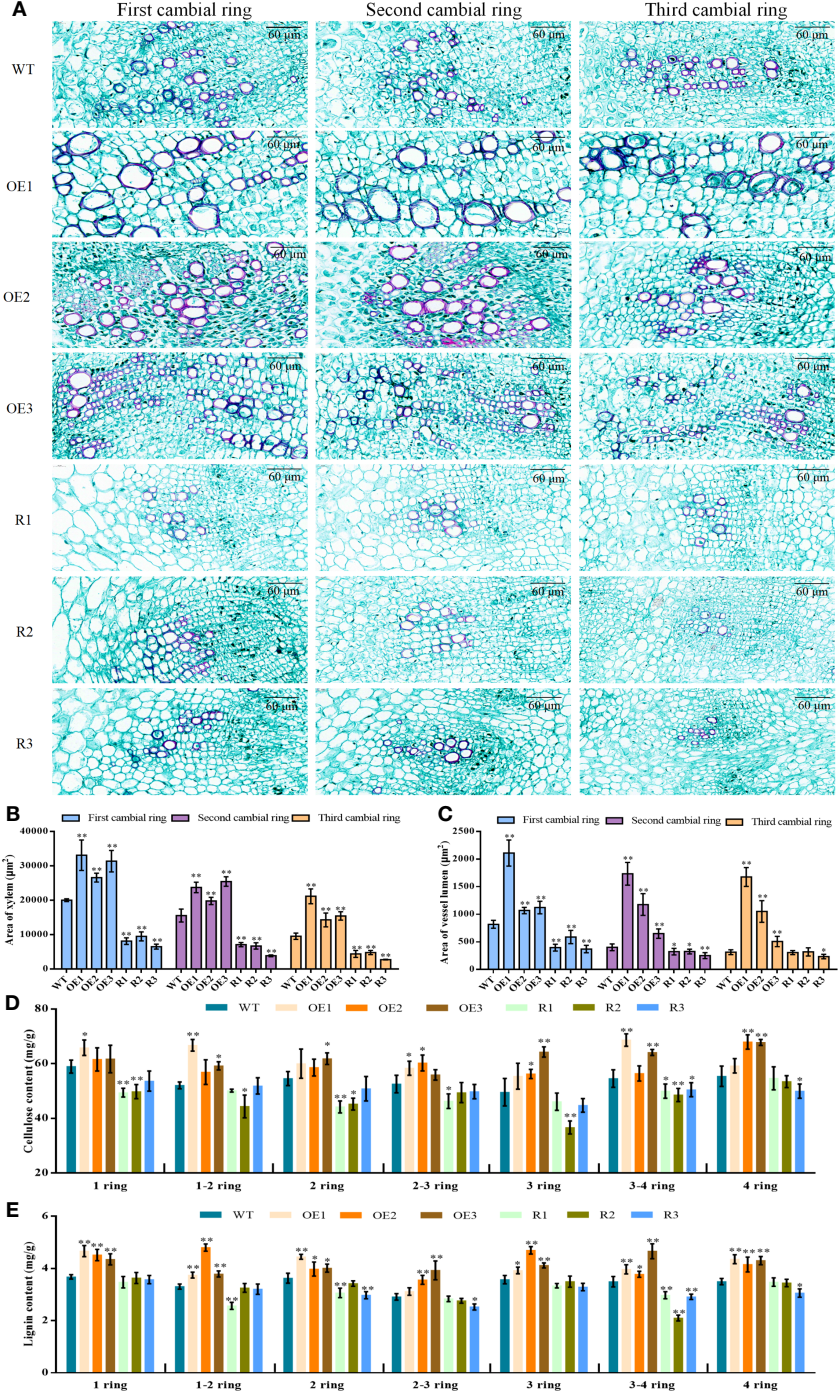
Xylem structure of the first, second, and third formative layers per unit area for each line. **(A)** Xylem structure of the first, second, and third formative layers. **(B)** Area of xylem in taproot 1,2,3 cambium. **(C)** Area of vessel lumen in taproot 1,2,3 cambium. **(D)** Cellulose content in taproot 1–4 cambium. **(E)** Lignin content in taproot 1–4 cambium. _*_ Student’s-t test, P < 0.05; _**_ Student’s-t test, P < 0.01. Scale bars = 60 μm.

To discover structural changes in cambial rings, we used CaseViewer to calculate the area of the xylem on the first, second, and third lamellar rings of beet taproot, and the size of the lumen area of the vessel on the xylem. The size of the xylem per unit area decreased sequentially from the first to the third layer for both the *BvCPD* transgenic and WT lines (**Figure 8A**). In each formation ring, the area of xylem in *BvCPD* overexpression lines was significantly larger than that of WT, while the development of xylem was inhibited and the area was significantly smaller in RNAi silencing lines (**Figure 8B**). It is worth noting that the reduction of the 1, 2, and 3 formative rings in the overexpression lines is not as significant as the WT. This indicates that *BvCPD* promotes the development of xylem, and the 2 and 3 rings are the most significant.

From microscopic observations, in the first, second, and third formative layers, ducts of the transgenic overexpression lines were all thicker compared with those of WT, while those of RNAi lines were slightly thinner compared with those of WT (**Figure 8A**). The vessel lumen areas of *BvCPD* overexpression lines were significantly higher compared with those of WT on the first, second, and third formative rings, while those of silenced lines were significantly lower compared with those of WT. There were highly significant differences for the first formative ring, but the differences gradually decreased in the second and third rings (**Figure 8C**). *BvCPD* overexpression lines had increased ductal pore lumen areas on the xylem of rings 1, 2, and 3, and the effect became more pronounced as the number of rings increased. The effect was more pronounced in the first and second formative loops of RNAi-interfering lines but weakened as the number of loops increased. In summary, overexpression of *BvCPD* was able to promote ductal development.

To assess whether *BvCPD* affects the composition of parenchyma cells and xylem in sugar beet taproot, cellulose and lignin contents in the 1–4 rings of each transgenic lines were measured. Cellulose and lignin contents were higher in overexpression lines than in WT (**Figures 8D, E**), but lower in silent lines compared with WT. This result shows that *BvCPD* affected the content of cellulose and lignin in 1–4 rings.

## 4 Discussion

C-3 oxidase, 5 α reductase, C-22 hydroxylase, C-23 hydroxylase, and C-6 oxidase are key enzymes in the BR synthesis pathway. *CPD* plays an important role as a C-3 oxidase in multiple steps of the BR synthesis pathway. Our results of subcellular localization revealed that BvCPD acts on the nucleus, cell membrane and cell wall. It was expressed in the taproot, petiole, and leaf of sugar beet, and was strongly expressed in the vascular bundles of various organs and parenchyma cells of taproot. This is slightly different from the other research. PeCPD of poplar was expressed in the endoplasmic reticulum in addition to being localized in the cell membrane (Wu et al., 2013). OsCPD1 and OsCPD2 of rice were both expressed in the endoplasmic reticulum (Zhan et al., 2022). Furthermore, AtCPD was strongly expression restricted to procambial cells of *Arabidopsis* roots when expressed under its natural promoter (Vukašinović et al., 2021). This indicates that the role of *CPD* in different plants also varies. The results of this study suggest that the expression of BvCPD in the nucleus, cell membrane, and cell wall may regulate cell wall relaxation, enhance the relative stability of the internal environment of cells, affect the development and differentiation of vascular bundles and parenchyma cells, and thus promote the expansion of sugar beet taproot. This is similar to the function of the poplar *PeCPD* gene, which regulates vascular development growth in *Arabidopsis thaliana* (Wu et al., 2013). Overexpression of *BvCPD* in sugar beet promotes BR synthesis, especially in roots. Unlike IAA and GA, BR lacks long-distance transport and acts only near the site of synthesis (Symons et al., 2008). Widespread expression of *BvCPD* in various tissues and organs may be more effective in maintaining local BR biosynthesis or gradients for optimal growth and development. The *CPD* gene in cotton and soybean both rescue root development, leaf expansion, and plant conformation in *cpd Arabidopsis* mutants (Wang et al., 2015; Liu et al., 2021). This was verified based on the observations in sugar beet *BvCPD* overexpressed and silenced plants.

*BvCPD* affected the morphological growth of sugar beet, especially the radial growth of the taproot. Different overexpression lines were taller than WT plants, with wide and long leaves, stout petioles, well-developed root systems, and clusters of lateral roots. Silent lines were shorter than WT, with short and narrow leaves and weak petioles. Further, individual lines also showed severe leaf curling and tiny root systems with almost no lateral roots. Notably, we found that *BvCPD* did not have a significant effect on the length of the taproot; however, it had a significant impact on the diameter of the taproot. When the *Arabidopsis bri1* mutant was disturbed by BR signaling, both radial and lateral growth were reduced, but the reduction in radial growth was less than that in lateral growth (Fridman et al., 2021). This suggests that *BvCPD* plays an important role in regulating the radial growth of sugar beet roots. Low concentrations of BR induce lateral root formation, but for lateral root formation, BR and growth hormone have a synergistic effect—a process that may be partially mediated by phospholipase A (Bao et al., 2004). *BvCPD* is significantly upregulated during the taproot growth and sucrose accumulation stages in beet taproot (Zhang et al., 2017), indicating that *BvCPD* is associated with beetroot development. The evidence that BR autonomously promotes root growth in *Arabidopsis thaliana* also supports the idea that *CPD* can regulate plant root growth (Müssig et al., 2003). The most significant differences in the relative expression of *BvCPD* and content of BR were found in the root (as compared with the petiole and leaf of transgenic sugar beet), with corresponding changes in levels of IAA and GA. *BvCPD* increased the endogenous content of BR and enhanced BR signaling in sugar beet while downregulating other synthetic genes such as *BvDET2*, *BvROT3*, and *BvBR6OX1*. This is in general agreement with the results of Wu et al. (2013) and Zheng et al. (2018).

We speculated that overexpression of *BvCPD* would also mimic the high level of BR in sugar beet and produce feedback inhibition of BR-related synthase genes, thereby maintaining normal levels of Campestanol in the plant (Simona et al., 2002). Studies on *Arabidopsis* showed that the expression of *AtDWF4, AtCPD, AtROT3*, and *AtCYP90D1* is negatively regulated by the BR signaling transcription factor *AtBZR1/2* (He et al., 2005). Exogenous BR treatment inhibits the expression of *AtDWF4*, *AtCPD*, *AtROT3*, and *AtCYP90D1* through *AtBZR1/2* (Goda et al., 2004; Ohnishi et al., 2006). This study found a similar BR negative feedback regulation mechanism in sugar beets. *BvBZR1* expression was upregulated to balance increased BR content *in vivo*, and *BvBZR1* reduced the expression of BR synthesis-related enzyme genes *BvDET2* and *BvROT3* through negative feedback regulation. *BvBR6OX1* is located downstream of *BvCPD* in the synthesis pathway, and it catalyzes the CS to BL transition. Unlike the two BR synthesis genes described above, *BvBR6OX1* was downregulated in expression in both the overexpression and RNAi-silenced lines compared with WT. We hypothesized that in the overexpression lines, as in the other synthetic genes, negative feedback regulation represses *BvBR6OX1* gene expression, owing to increased BR content. However, silent lines may not be affected by changes in BR content, but only by the disrupted expression of *BvCPD*, which generates less substrate after catalysis, ultimately leading to significant downregulation of *BvBR6OX1*. The GA biosynthetic pathway is linked to the BR signaling pathway via *BZR1* and *DELLA* (Li et al., 2012). In addition, earlier studies reported that BRs could induce the expression of GAs synthesis genes (Bouquin et al., 2001). In this study, enhanced BR signaling caused upregulated expression of *BvTAA1* and *BvGA3OX1*, stimulating the synthesis of IAA and GA. All three hormones were significantly increased in overexpressed sugar beet taproot. BR, IAA, and GA may act synergistically to affect sugar beet root growth and lead to taproot expansion. However, the specific regulatory mechanisms still need to be elucidated.

*BvCPD* promotes the division and expansion of parenchyma cells between the rings of the sugar beet taproot; additionally, it also promotes the development of the xylem of the vascular bundle and the lumen of the vessel. The synergy of the two factors makes the sugar beet taproot develop rapidly and increases the diameter of the taproot. In this study, *BvCPD* caused changes in endogenous hormones to produce changes in sugar beet root morphology. Therefore, cross-sectional taproot paraffin sections were made for the transgenic lines and WT. The first four rings on the taproot were observed. The ring spacing, number of cell layers, size of parenchyma cells, xylem area, and area of vessel lumen of *BvCPD* overexpressing lines were significantly larger than those of WT, whereas all indicators of silent lines were smaller. This indicates that *BvCPD* promotes BR synthesis and may exert synergistic effects with IAA and GA. This increases the size and number of parenchyma cells and the spacing between rings. *HAT7* and *GTL1* are regulatory factors induced by BR along cortical trajectories, controlling cell extension (Nolan et al., 2023). Exogenous BR treatment can induce the expression of cell Cyclin D3 to regulate cell division in roots (González-García et al., 2011). After enhanced BR signal in cotton, the transcription factor GhBES1.4 regulates the downstream target gene *GhKRP6*, causing it to interact with the G-type CDK protein *GhCDKG* that affects cell cycle splicing, ultimately regulating cell proliferation (Gu et al., 2023). We speculate that the increase or decrease in BR content caused by *BvCPD* in transgenic sugar beet strains will also regulate such genes, affecting the expansion of parenchyma cells in the taproot.

Moreover, BR accelerates the transport of water and nutrients by promoting the development of the xylem and ducts. Therefore, the beet experiences strong physiological metabolism that accelerates growth and development. The reason is that BR can promote cell wall growth by regulating genes associated with cell wall biosynthesis, such as *BvXTH33*, *BvSHV3*, *BvCESA6*, *BvPARVUS*, and *BvCEL1*, and it can also promote secondary xylem development (Wang et al., 2021). The BR biosynthetic mutant *cpd*, the BR receptor mutant *bri1*, and the BR signaling mutant *bin2* all produced fewer vascular bundles. In contrast, overexpression of the *BRI1* and BR signaling genes in plants results in more vascular bundles (Ibañes et al., 2009). Overexpression of *BvCPD* between and on different rings also promotes the accumulation of cellulose and lignin, the main components of the cell wall, which provides a reliable basis for the development of vascular bundles. The relative expression of *PbrMYB24* in pear flesh is significantly positively correlated with lignin and cellulose content. *PbrMYB169* and *PbrNSC* can activate the promoter of *PbrMYB24*, enhancing gene expression (Xue et al., 2023). The *LAC4* and *LAC17* genes can also affect the content of lignin, especially the silencing of *LAC17*, which specifically affects the deposition of G–lignin units in fibers (Serge et al., 2011). Therefore, future research should focus on the mechanism of action of *BvCPD* in the growth of sugar beet taproot, and identify genes that are regulated to cause changes in cellulose and lignin content.

**Figure 9:**
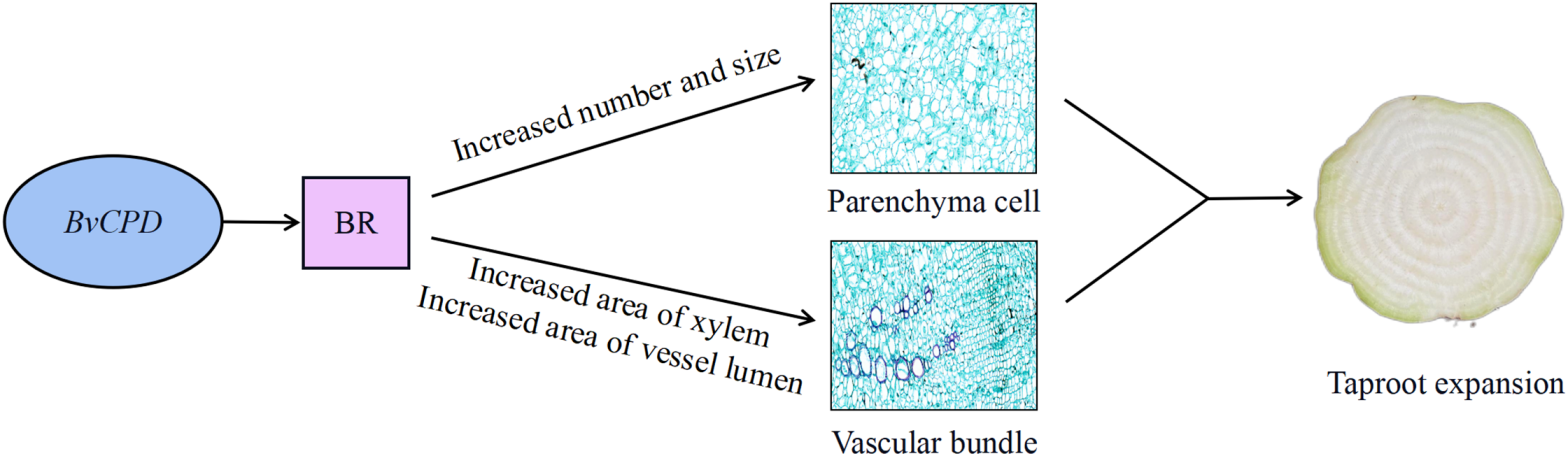
Hypothetical model of *BvCPD* roles in taproot expansion of sugar beet. Overexpression of *BvCPD* in taproot increases BR levels and induces changes in parenchyma cells and vascular bundles to promote taproot expansion. Arrows indicate positive effects.

In summary, *CPD* plays a major role in root development. Overexpression of *BvCPD* in taproot increases BR levels and upregulates *BvBZR1* expression. *BvBZR1* regulates *BvTAA1* and *BvGA3OX1* to increase IAA and GA contents. We speculate that the three hormones act synergistically to induce changes in parenchyma cells and vascular bundles to promote taproot expansion (**Figure 9**). Reduced *BvCPD* expression restricts taproot expansion. This study provides direct genetic evidence that *BvCPD* is a functional gene for BR synthase in sugar beet and affects the lateral root growth of beet. Our results provide a theoretical basis for sugar beet root expansion study.

## 5 Conclusions

*BvCPD* promotes the synthesis of BR and causes changes in endogenous IAA and GA contents. This increases taproot diameter by regulating the number and size of parenchyma cells and areas of the xylem and vessel lumen of vascular bundles, leading to the expansion of sugar beet taproot. This is the first report to identify the function of *CPD* in sugar beet and provides a basis for further studies on the mechanism of action of *CPD* in sugar beet.

## Data availability statement

The original contributions presented in the study are included in the article/**Supplementary Files**, further inquiries can be directed to the corresponding authors.

## Author contributions

XG: Data curation, Writing – original draft, Writing – review & editing. YL: Writing – review & editing. NL: Writing – review & editing. GL: Writing – review & editing. YS: Writing – review & editing. SZ: Project administration, Writing – review & editing.
