# Supplementary material for: BvCPD promotes parenchyma cell and vascular bundle development in sugar beet (Beta vulgaris L.) taproot
Source: Front Plant Sci. 2023 Sep 12;14:1271329. doi: 10.3389/fpls.2023.1271329 (PMC10523326; doi:10.3389/fpls.2023.1271329)
Supplement: Supplementary file 1 [file DataSheet_1.pdf]

Supplementary Material

BvCPD promotes parenchyma cell and vascular bundle development in sugar beet (Beta vulgaris L.) taproot

Xiao-Tong Guo, Yue Li, Ning-Ning Li, Guo-Long Li, Ya-Qing Sun*, Shao-Ying Zhang*

***Correspondence:** Shao-Ying Zhang: syzh36@aliyun.com (SY.Z.), Ya-Qing Sun:syaqing@imau.edu.cn (YQ.S.)

1 Supplementary Tables

Supplementary Table S1. Probe information

CPD-ORF mix
5´-CATTGAGTGAAGGGCATTGATTTGT-3´
5´-ATGCCTCCGATGATATTTGCGACT-3´
5´-TAGCCACTAATGTGAACGTCTGATATA-3´

Supplementary Table S2. Medium formulation

Name	Formulation
Germination medium	1 L Tap water + 1 mg/L 6-BA + 8.0 g/L Agar, pH = 5.8
Pre-culture medium	MS + 30 g Sucrose + 1 mg/L 6-BA + 0.2 g Casein + 7 g/L Agar, pH = 5.8
Co-culture medium	MS + 30 g Sucrose + 1 mg/L 6-BA + 0.2 g Casein + 100 μM AS + 7g/L Agar, pH = 5.8
Suspension medium	1/2MS + 50 g Sucrose + 5g MES + 0.02% Silwet-77, pH = 5.8
Inhibition medium	MS + 30 g Sucrose + 0.25 mg/L NAA + 1 mg/L 6-BA + 0.5 mL/L PPM + 8.0 g/L Agar + 400 mg/mL Temetin, pH = 5.8
Screening medium	MS + 30 g Sucrose + 0.25 mg/L NAA + 1mg/L 6-BA + 0.5 mL/L PPM + 8.0 g/L Agar + 6 mg/mL Hygromycin, pH = 5.8
Differentiation medium	MS + 30 g Sucrose + 0.25 mg/L NAA + 1 mg/L 6-BA + 0.5mL/L PPM + 8.0 g/L Agar, pH = 5.8
Rooting medium	MS + 30 g Sucrose + 2 mg/L NAA + 0.5 mL/L PPM + 8.0 g/L Agar, pH = 5.8

Supplementary Table S3. Primer sequences used for qRT–PCR

Gene ID	Forward primer (5´→3´)	Reverse primer (5´→3´)
BvCPD-OE	CGGGATCCTCCTCAAACTCAACTTTC CCCT	ACGCGTCGACGTTCACATCCCTCGTTTTC CTCT
BvCPD-RP	CGGGATCCCGGACGAGATGATAGTGG ATTTCC	GACTAGTCTACGCCTCTAAATGCCGCAA AC
BvCPD-RA	CGAGCTCGTACGCCTCTAAATGCOGC AAAC	GGGGTACCCCGACGAGATGATAGTGGAT TTCC
BvCPD-G	CTCTTGACGAGCTCGCTCTCTCCTCAA ACTCAACTTTCCC	TGCTCACCATGTCGAGCACGTAAATGGG GTATCGC
HYG-2	GTTTAGCGAGAGCCTGACCT	GTCGTCCATCACAGTTTGCC
BvCPD-EXP	TGGCTTGGTGGTAAAGGAGAG	GAGTCATAGTTGTGGAGGTGGT
BvBZR1	TGGATTGTTGAGCCTGATGGT	GCTTTGTGGAGCAGAAGAAGAG
BvDET2	TACCCGACAGCCCTCCTAAA	CCCACCCTGAACGAAAATGG
BvROT3	TGGGTCCTGGTGAAGAAATGG	ATGTTGCTATGTTGCTCGTGTC
BvBR6OX1	TGTTGTAGGGACTCTTTCGCT	CACTATCACCTTCCTTGCCTG
BvTAA1	CCATCACACAGAATCGGCACA	TGGCGTAGCAGAAACAACACT
BvGA3OX1	TTTGATGCCACACACAGACAG	TACACACCATTGCTCCAAGCC
BvACTIN	TGCTTGACTCTGGTGATGGT	AGCAAGATCCAAACGGAGAATG

2 Supplementary Figures

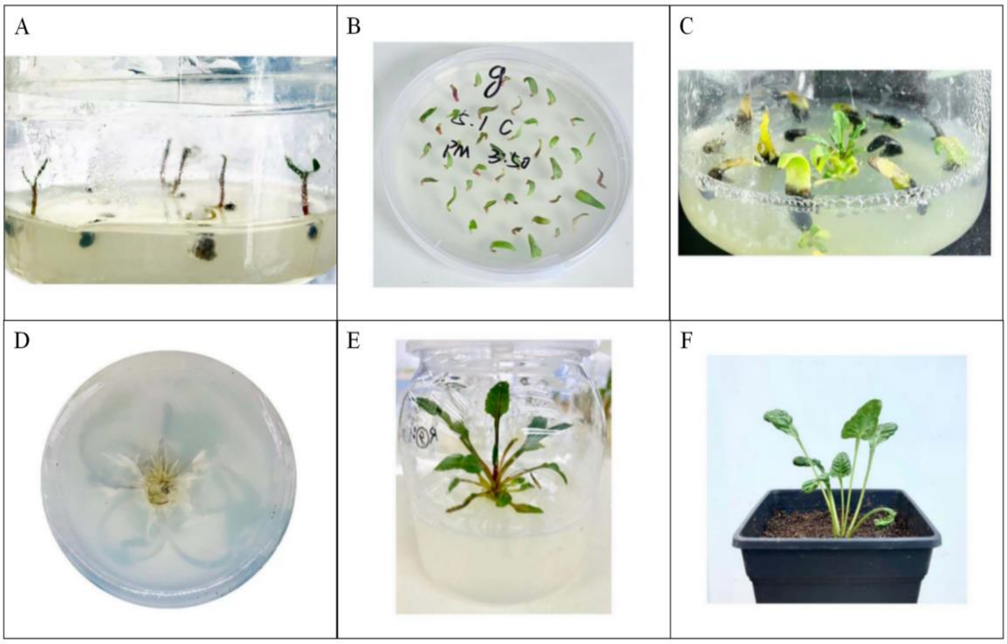

Supplementary Figure S1. Genetic conversion process of sugar beet. **(A)** Beets without bacterial seedlings. **(B)** Sweet beet leaf knot after agrobacterium infection. **(C)** Positive plants screened for hygromycin. **(D)** Root culture of transgenic plants. **(E)** Transgenic plants. **(F)** Transplantation of transgenic plants.
